# Low Circulating Levels of Omentin-1 and Irisin in Type 2 Diabetes Mellitus Patients with Metabolic-Associated Fatty Liver Disease

**DOI:** 10.5152/tjg.2025.24639

**Published:** 2025-03-17

**Authors:** Hua-Ying Li, Yan-Yan Zhang, Xia-Ming Cai, Xiang Chen

**Affiliations:** 1 Department of Endocrinology, the Affiliated Hospital of Putian University, Fujian, China; 2 Clinical Laboratory, the Affiliated Hospital of Putian University, Fujian, China

**Keywords:** Irisin, metabolic-associated fatty liver disease, omentin-1, type 2 diabetes mellitus, waist-to-hip ratio

## Abstract

**Background/Aims:**

The study analyzed the roles of circulating omentin-1 and irisin in patients with type 2 diabetes mellitus (T2DM) concomitant with metabolic-associated fatty liver disease (MAFLD).

**Materials and Methods:**

This cross-sectional study included 80 patients with T2DM but no MAFLD, 62 patients with MAFLD but no T2DM, 50 T2DM patients having MAFLD (T2DM/MAFLD), and 80 healthy individuals.

**Results:**

The serum levels of omentin-1 and irisin were both significantly reduced in patients with T2DM coexisting with MAFLD compared to T2DM or MAFLD patients alone. In T2DM patients, the level of omentin-1 decreased as the level of fasting plasma glucose (FPG) increased and the level of high-density lipoprotein cholesterol (HDL-C) reduced; the level of irisin decreased as the levels of FPG and fasting insulin (FINS) increased. In MAFLD patients, a lower level of omentin-1 was correlated with a lower level of HDL-C but with a greater waist-to-hip ratio (WHR), alanine aminotransferase and aspartate aminotransferase levels; a lower level of irisin was correlated with higher WHR and FINS level. In patients with T2DM coexisting with MAFLD, those with a lower level of omentin-1 were found to have a lower level of HDL-C concurrent with lower WHR and triglyceride level; and those with a lower level of irisin showed lower WHR, FPG and FINS levels. Combined evaluation of omentin-1 and irisin for diagnosing T2DM coexisting with MAFLD yielded an area under the curve of 0.943.

**Conclusion:**

These findings suggest the assessment potential of omentin-1 and irisin for T2DM coexisting with MAFLD.

**Main Points**

- The serum levels of omentin-1 and irisin were decreased in patients with type 2 diabetes mellitus (T2DM) concomitant with metabolic-associated fatty liver disease (MAFLD).
- The serum levels of omentin-1 and irisin were similar in patients with T2DM or MAFLD alone.
- The receiver operating characteristic curves of omentin-1 and irisin as a combined tool in diagnostic tests for T2DM and MAFLD alone or T2DM associated with MAFLD produced AUC values of 0.810, 0.839, and 0.943, respectively.

## Introduction

Metabolic-associated fatty liver disease (MAFLD), a novel nomenclature replacing non-alcoholic fatty liver disease, is the hepatic component of metabolic disorder, showing the increasing incidence of obesity and type 2 diabetes mellitus (T2DM) in many countries.^1^^,^^2^ Metabolic-associated fatty liver disease shares a bidirectional association with T2DM due to similar disease mechanisms and affects nearly 55% of people living with T2DM.^3^ The coexistence of these 2 disorders is more likely to potentiate the disease-related outcomes and increase the incidence of complications of the individual diseases, which is often overlooked by both diabetologists and hepatologists. Therefore, biomarker profiles to identify the coexistence of T2DM and MAFLD and rapid progression of the disease are urgently needed to improve, and, importantly, to personalize management.

Omentin-1 is an adipokine belonging to 1 of 2 isoforms (1 and 2) of omentin that is predominantly produced by visceral fat tissue, specifically secreted from the stromal vascular fraction cells of white adipose tissue.^4^ Although omentin-1 and omentin-2 are both functionally expressed in visceral fat tissue, omentin-1 is the major circulating isoform.^5^ Omentin-1 has been extensively studied not only for its favorable effects on glucose homeostasis and insulin metabolism in metabolic disorders, but also for its atheroprotective and anti-inflammatory effects in cardiovascular diseases.^6^ Circulating omentin-1 is decreased in overweight and obese subjects but its level is elevated after weight loss or intake of antidiabetic drugs.^7^^,^^8^ Reduced omentin-1 has been found to be associated with the rapid progression of MAFLD.^9^ Irisin is a recently identified myokine that increases thermogenesis-associated energy expenditure and functions as a linkage between muscles and other tissues to brown white adipose tissue and regulate metabolic processes.^10^ An increase in circulating irisin may contribute to improved glucose homeostasis by preventing insulin resistance.^11^ Circulating irisin has been shown to decline in obese patients and patients with nonalcoholic simple steatosis and steatohepatitis compared to healthy individuals.^12^ A recent study has demonstrated increases in omentin-1 and irisin levels in the circulation of patients with diabetic foot ulcer after surgery for ulcer wound closure, indicating roles for omentin-1 and irisin in metabolic disorders with rapid progression.^13^ Most earlier studies focus on the relationship of omentin-1 and irisin with the presence of T2DM or MAFLD in the general population, but it remains unknown whether omentin-1 and irisin can be 2 determinants in the context of MAFLD coexisting with T2DM. Accordingly, we proposed an interesting hypothesis that serum levels of omentin-1 and irisin differ more significantly in patients with MAFLD and T2DM together compared to T2DM or MAFLD alone. To prove this hypothesis, we recruited patients with T2DM but no MAFLD, those with MAFLD but no T2DM, those with both diseases together, and healthy individuals to determine serum levels of omentin-1 and irisin. Additionally, possible relationships with anthropometric and metabolic parameters were investigated.

## Materials and Methods

### Study Population

This cross-sectional study included 80 patients with T2DM but no MAFLD, 62 patients with MAFLD but no T2DM, 50 patients with T2DM complicated with MAFLD (T2DM/MAFLD), and 80 healthy individuals. The recruitment occurred between October 2022 and December 2023. All included participants must be aged 18 years or above. Healthy individuals were included during physical examinations proving their healthy conditions, especially with no metabolic disorders. Patients were included for a new diagnosis of T2DM conforming to the American Diabetes Association (ADA) criteria.^14^ Patients were included for a new diagnosis of MAFLD confirmed by 2 or more of the following abdominal ultrasonographic findings: i) liver echogenicity in the near field enhanced compared to the kidney or spleen; ii) unclear intrahepatic tube structure; and iii) deep attenuation of liver echogenicity in the far field.^15^ The recruitment applied the exclusion criteria as follows: other types of diabetes (type 1 diabetes, gestational diabetes mellitus, diabetic ketoacidosis, or hyperglycemic hyperosmolar state), acute complications of diabetes, receiving any diabetes treatment, substantial alcohol use (women: alcohol consumption >70 g per week; men: alcohol consumption >140 g per week; or >40 g per day for more than 5 years),^16^ hepatitis virus infection, autoimmune liver disease or drug-induced damage to the liver, acute or chronic renal failure, thyroid dysfunction, infectious diseases, Wilson’s disease, congenital cardiac disease, malignant tumors, or mental diseases. The study protocol complied with the principles of the Declaration of Helsinki and was approved by the Ethics Committee of the Affiliated Hospital of Putian University (approval no: 2024199, date: November 8, 2024). All participants signed the informed consent form prior to recruitment.

### Definitions

Metabolic-associated fatty liver disease was defined based on the proposed criteria by a panel of international experts from 22 countries^17^^,^^18^ as imaging evidence of hepatic steatosis with the coexistence of T2DM, overweight/obesity,^19^ or ≥2 metabolic abnormalities: i) for Asian men and women (central obesity), waist circumference (WC) ≥90/80 cm; ii) blood pressure ≥130/85 mm Hg or use of antihypertensives; iii) plasma triglyceride (TG) ≥1.7 mmol/L; iv) plasma high-density lipoprotein cholesterol (HDL-C) <1.03/1.29 mmol/L for men/women; v) prediabetes, such as 5.6-6.9 mmol/L of fasting plasma glucose (FPG) and 5.7%-6.4% of glycohemoglobin; vi) homeostasis model assessment of insulin resistance (HOMA-IR) ≥2.5; and vii) plasma high-sensitivity C-reactive protein level >2 mg/L.

### Anthropometric and Biochemical Measurements

Anthropometric parameters included age, sex, height (in meters), weight (in kilograms), body mass index (BMI), WC, hip circumference (HC), systolic blood pressure (SBP), diastolic blood pressure (DBP), and duration of DM. Height and weight were measured with the patients wearing light clothes and no shoes. Body mass index equals the ratio of weight to squared height, namely kg/m^2^. The WC was measured as the circumference, in the horizontal plane, at the mid-point between the iliac crest and the lower rib margin using a 150-cm flexible tape. The HC was measured at the widest part of the buttocks in a standing position using a 150-cm flexible tape. The waist-to-hip ratio (WHR) was calculated by dividing WC by HC. Systolic blood pressure and DBP were measured after 15 minutes rest in a sitting position with a manual sphygmomanometer. Biochemical parameters included fasting plasma glucose (FPG), total cholesterol (TC), low-density lipoprotein cholesterol (LDL-C), HDL-C, TG, alanine aminotransferase (ALT), aspartate aminotransferase (AST), and gamma-glutamyl transferase (GGT). The levels of FPG, TC, LDL-C, HDL-C, and TG were measured with the aid of the Beckman Coulter AU5800 fully automated biochemical analyzer (Beckman Coulter, Inc., Fujian, China). The fasting ALT level was determined by the colorimetric method by using the ALT Assay Kit (MAK055, Sigma-Aldrich, St. Louis, MO, USA). The fasting AST level was determined by the colorimetric method by using the AST Assay Kit (MAK467, Sigma-Aldrich). The GGT level was determined by the colorimetric method by using the GGT Activity Assay Kit (E-BC-K126-M, Elabscience, Wuhan, China). Fasting insulin (FINS) was determined by the electrochemiluminescence immunoassay method using Roche Diagnostics kits (Roche Diagnostics GmbH, Mannheim, Germany). Insulin resistance was reflected by the HOMA-IR index, which was obtained by the equation: FPG (mmol/L) × FINS (mLU/mL) / 22.5.

### Circulating Omentin-1 and Irisin Assessment

Peripheral blood samples (5 mL) were collected from each participant after 10-12 hours of overnight fasting and underwent centrifugation (3000 × *g*) for 10 minutes to separate the serum. The serum was frozen at −80°C. The serum samples were analyzed using commercially available human ELISA kits for omentin-1 (Cat. no. DY4254-05, R&D Systems, USA) and irisin (Cat. DY9420-05, R&D Systems, USA) according to the kits’ instructions. Briefly, each well in the microplate was added with 100 μL Assay Diluent and the same volume of omentin-1 and irisin standards or samples. After incubation for 2 hours at room temperature and 3 washes, the microplate was treated with peroxidase-conjugated anti-omentin-1 antibody or anti-irisin antibody (200 μL per well). After another 2-hour incubation and 3 more washes, the microplate reacted with peroxidase-specific substrate (200 μL per well) for 20 minutes. By adding 50 μL 2 N sulfuric acid for each well, the peroxidase reaction in the microplates was terminated. For the omentin-1 kit, the assay range was 187.0-12 000 pg/mL, with the inter- and intra-assay coefficients of variation (CVs) both <10%. For the irisin kit, the assay range was 250.0-8000 pg/mL, with the inter- and intra-assay CVs both <10%.

### Statistical Analysis

Sample size calculation was computed with prior power analysis using the G*power software version 3.1.9.2 (Heinrich-Heine-Universitt Düsseldorf, Düsseldorf, Germany) setting effect size as 0.25, *α* as 0.05, power as 0.9, and number of groups as 4, with a loss of 10%. We performed data analysis and figure visualization with the aid of SPSS version 20 (IBM SPSS Corp.; Armonk, NY, USA). Results included qualitative variables and quantitative variables. The qualitative variables were summarized as percentages. After Shapiro–Wilk tests, the quantitative variables normally distributed were summarized as mean ± standard deviation (SD) and those non-normally distributed were summarized as median with range. The statistical differences of quantitative variables normally distributed among T2DM, MAFLD, T2DM/MAFLD, and control groups were examined by using 1-way analysis of variance (ANOVA) with Tukey’s multiple comparisons test, and the statistical differences of those non-normally distributed were examined by the Kruskal–Wallis test with Dunn’s multiple comparisons test. Fisher’s exact test was carried out for qualitative variables. Pearson’s correlations were employed to identify the correlation between circulating omentin-1 and irisin levels, and significant anthropometric and biochemical variables among T2DM, MAFLD, and T2DM/MAFLD groups for quantitative variables normally distributed, and Spearman’s correlations were employed for quantitative variables non-normally distributed. A prediction model using receiver operating characteristic (ROC) curves and its summary statistics [area under the curve (AUC)] was analyzed in the target population (T2DM, MAFLD, and T2DM/MAFLD groups) differing from the source population (control group).

## Results

### Anthropometric Characteristics and Clinical/Biochemical Profiles

A total of 272 participants fulfilling the inclusion and exclusion criteria were recruited into the study, consisting of 80 patients with T2DM but no MAFLD, 62 patients with MAFLD but no T2DM, 50 patients with T2DM complicated with MAFLD (T2DM/MAFLD), and 80 healthy individuals. Table 1 summarizes their anthropometric characteristics and clinical/biochemical profiles. From this table, we can observe no significant difference regarding individuals’ age, sex distribution, and TC levels among T2DM, MAFLD, T2DM/MAFLD, and control groups. The BMI, ALT, AST, GGT levels, and WHR were increased in the MAFLD and T2DM/MAFLD groups compared to the T2DM and control groups (*P* < .05). Metabolic-associated fatty liver disease and T2DM/MAFLD groups did not differ in these parameters (*P* > .05), between T2DM and control groups (*P* > .05), except WHR, which was remarkably lower in the MAFLD group than the T2DM/MAFLD group (*P* < .05). The T2DM/MAFLD groups had increased SBP and DBP compared to the control group (*P* < .05). Among T2DM, MAFLD, and T2DM/MAFLD groups, only a 2-group difference in the SBP value was noted: MAFLD group vs. T2DM/MAFLD group (*P* < .05). The T2DM group showed a higher SBP than the control group (*P* < .05). The FPG level and HOMA-IR were higher in the T2DM and T2DM/MAFLD groups than the MAFLD and control group (*P* < .05). The 3 disease groups displayed elevated FINS levels compared to the control group (*P* < .05). Among T2DM, MAFLD, and T2DM/MAFLD groups, only a 2-group difference in the FINS level was noted: T2DM group vs. T2DM/MAFLD group (*P* < .05). The MAFLD group had a raised level of LDL-C compared to the T2DM and control groups. The 3 disease groups showed a decreased HDL-C level with an increased TG level compared to the control group (*P* < .05). The MAFLD and T2DM/MAFLD groups had a higher level of TG than the T2DM group (*P* < .05).

### Serum Levels of Omentin-1 and Irisin in Type 2 Diabetes Mellitus and/or Metabolic-Associated Fatty Liver Disease

**Figure 1.**
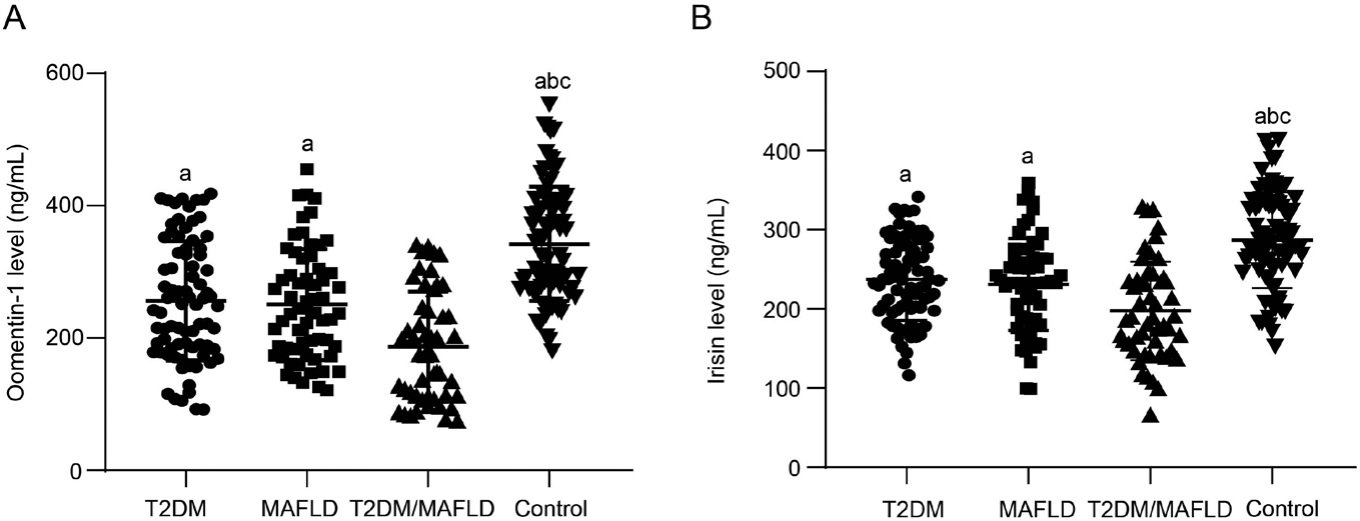
The serum levels of omentin-1 and irisin of participants with T2DM (n = 80), MAFLD (n = 62), T2DM/MAFLD (n = 50), and control (n = 80) groups determined by ELISA methods. A, Omentin-1 (ng/mL). B, Irisin (ng/mL).

The median values of omentin-1 level in T2DM, MAFLD, T2DM/MAFLD, and control groups were 251.0, 237.4, 175.8, and 322.4 ng/mL, respectively (Figure 1A). Significant differences were noted among the 4 groups (*P* < .05). The subsequent multiple comparisons test showed the T2DM/MAFLD group had the lowest omentin-1 level and the control groups had the highest level (*P* < .05), but a similar level showed between T2DM and MAFLD group (*P* > .05). The median values of irisin levels in T2DM, MAFLD, T2DM/MAFLD, and control groups were 237.0, 232.5, 187.7, and 284.5 ng/mL, respectively (Figure 1B). The level of irisin exhibited a similar pattern to that of omentin-1 among the 4 groups (*P* < .05). The T2DM/MAFLD group had the lowest irisin level, and the control groups had the highest level (*P* < .05), but a similar level showed between T2DM and MAFLD group (*P* > .05).

### Characteristics of Circulating Omentin-1 and Irisin in Type 2 Diabetes Mellitus and/or Metabolic-Associated Fatty Liver Disease

The correlation coefficient (*r* values) obtained by Pearson’s correlation or Spearman’s correlation tests to characterize the serum levels of omentin-1 and irisin in T2DM patients, MAFLD patients, and patients with T2DM coexisting with MAFLD are all presented in Table 2. In T2DM patients, the level of omentin-1 decreased as the level of FPG increased and the level of HDL-C reduced; the level of irisin decreased as the levels of FPG and FINS increased. In MAFLD patients, a lower level of omentin-1 was correlated with a lower level of HDL-C but with greater WHR, ALT, and AST levels; a lower level of irisin was correlated with higher WHR and FINS level. In patients with T2DM coexisting with MAFLD, those with a lower level of omentin-1 were found with a lower level of HDL-C concurrent with lower WHR and TG levels; and those with a lower level of irisin showed lower WHR, FPG and FINS levels. These results indicate that reduced circulating levels of omentin-1 and irisin may be associated with unfavorable anthropometric and metabolic parameters of T2DM patients, MAFLD patients, and patients with T2DM coexisting with MAFLD.

### Circulating Omentin-1 and Irisin as Tools to Diagnose Type 2 Diabetes Mellitus and/or Metabolic-Associated Fatty Liver Disease

**Figure 2.**
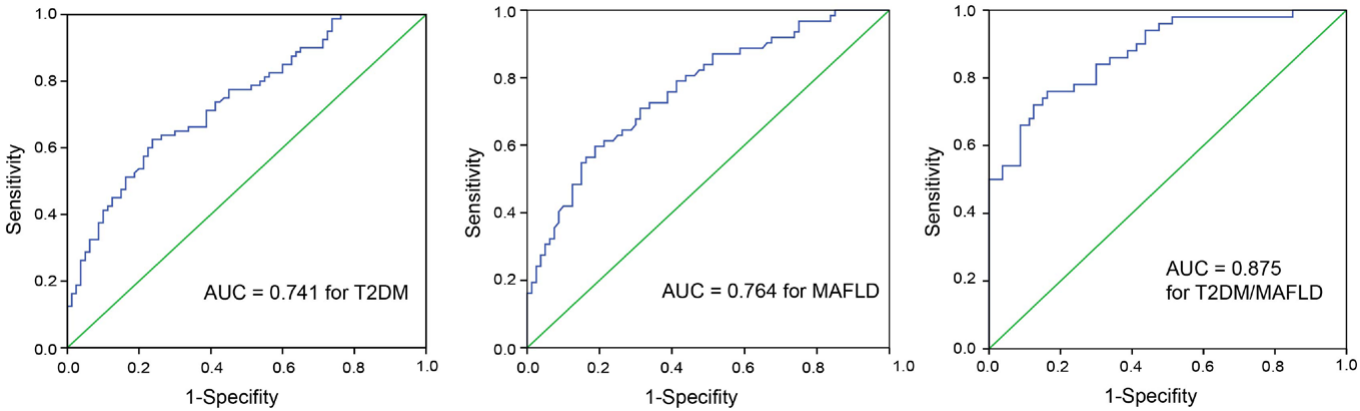
The ROC curve with AUC showing omentin-1 level (ng/mL) as a tool to diagnose T2DM patients among healthy individuals, MAFLD patients among healthy individuals, and patients with T2DM/MAFLD among healthy individuals.

**Figure 3.**
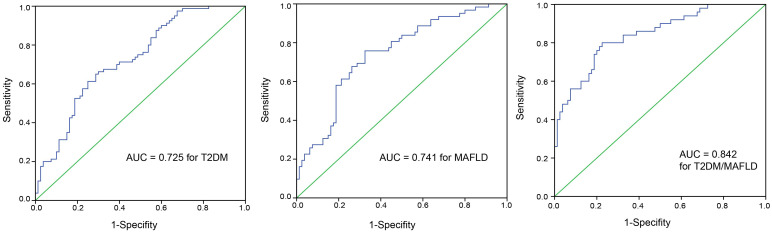
The ROC curve with AUC showing irisin level (ng/mL) as a tool to diagnose T2DM patients among healthy individuals, MAFLD patients among healthy individuals, and patients with T2DM/MAFLD among healthy individuals.

**Figure 4.**
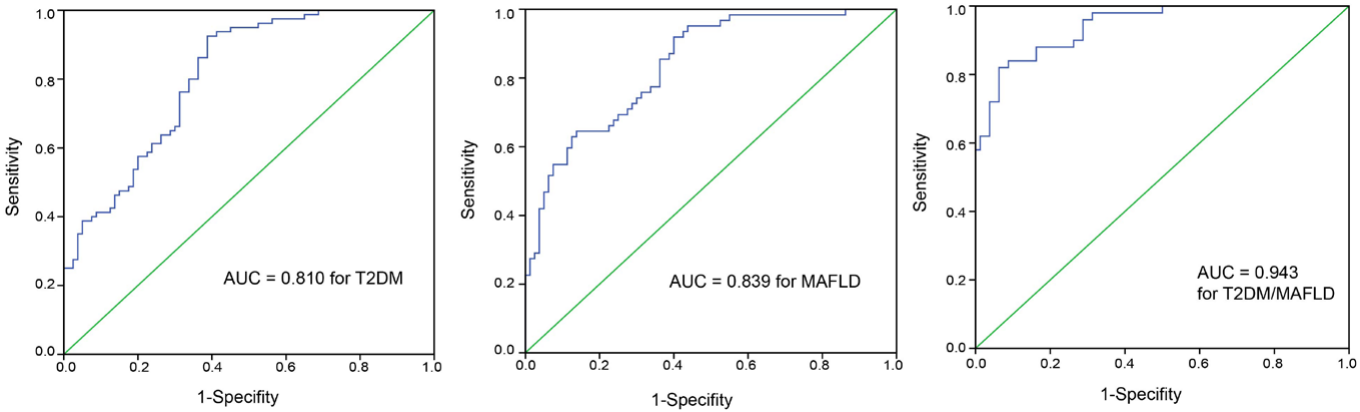
The ROC curve with AUC showing omentin-1 and irisin as a combined tool to diagnose T2DM patients among healthy individuals, MAFLD patients among healthy individuals, and patients with T2DM/MAFLD among healthy individuals.

The ROC curves of omentin-1 in diagnostic tests for T2DM, MAFLD, and T2DM/MAFLD populations are plotted in Figure 2. The performance of omentin-1 detection for T2DM was: AUC value, 0.741 (95% CI, 0.666-0.816); sensitivity, 0.63; and specificity, 0.77. The performance of omentin-1 detection for MAFLD was: AUC value, 0.764 (95% CI, 0.686-0.842); sensitivity, 0.62; and specificity, 0.79. The performance of omentin-1 detection for T2DM/MAFLD was: AUC value, 0.875 (95% CI, 0.814-0.936); sensitivity, 0.76; and specificity, 0.84. The ROC curves of irisin in diagnostic tests for T2DM, MAFLD, and T2DM/MAFLD populations are plotted in Figure 3. The performance of irisin detection for T2DM was: AUC value, 0.725 (95% CI, 0.647- 0.803); sensitivity, 0.62; and specificity, 0.75. The performance of irisin detection for MAFLD was: AUC value, 0.741 (95% CI, 0.660-0.823); sensitivity, 0.76; and specificity, 0.68. The performance of irisin detection for T2DM/MAFLD was: AUC value, 0.842 (95% CI, 0.773-0.911); sensitivity, 0.80; and specificity, 0.78. The ROC curves of omentin-1 and irisin as a combined tool in diagnostic tests for T2DM, MAFLD, and T2DM/MAFLD populations are plotted in Figure 4. The performance of combined detection for T2DM was: AUC value, 0.810 (95% CI, 0.745-0.876); sensitivity, 0.93; and specificity, 0.62. The performance of combined detection for MAFLD was: AUC value, 0.839 (95% CI, 0.775-0.902); sensitivity, 0.92; and specificity, 0.60. The performance of combined detection for T2DM/MAFLD was: AUC value, 0.943 (95% CI, 0.906-0.979); sensitivity, 0.82; and specificity, 0.94.

## Discussion

The current investigation demonstrated lower circulating omentin-1 and irisin in patients with either T2DM or MAFLD compared to lean controls. Further detections showed that those with T2DM coexisting with MAFLD exhibited even more decreased omentin-1 and irisin levels. The performance of omentin-1 and irisin as a combined tool in diagnostic tests for both diseases in 1 patient was better than when used alone, indicating omentin-1 and irisin may have dual implications in metabolic disorder with a bidirectional association.

The expression of omentin-1 in adipocytes is reduced by glucose/insulin, which is important for maintaining insulin sensitivity and body metabolism. Decreased omentin-1 levels have been closely associated with the incidence of T2DM and several complications, including diabetic vascular disease, cardiomyopathy, and retinopathy. Earlier experimental evidence has proven that the anti-inflammatory and cardiovascular protective effects of omentin-1 are achieved by modulating several signaling pathways involving AMP-activated protein kinase (AMPK), nuclear factor-κB, and mitogen-activated protein kinase (MAPK).^20^ Antidiabetic therapies, weight loss, aerobic training, and an olive oil-rich diet have been demonstrated to effectively increase circulating omentin-1 levels.^21^ Circulating omentin-1 levels were notably related to metabolic status in people with obesity, while no relationship was noted between omentin-1 and metabolic status in normal-weight people.^22^ This suggested the relationship between omentin-1 and metabolic diseases. Accordingly, a previous study demonstrated that circulating omentin-1 was inversely correlated with glucose levels in patients with MAFLD.^23^ Recent preclinical data have shown that supplementation with omentin-1 may inhibit the NF-κB and MAPK signaling pathways to maintain glucose and insulin metabolism in the context of metabolic dysfunction-associated steatotic liver disease.^9^ Our results that the study population of T2DM and MAFLD alone exhibited reduced circulating levels of omentin-1 compared to the general population. In coexistent populations, omentin-1 was found to exhibit a lower level than in single disease, such as in T2DM patients coexisting with peripheral artery disease and MAFLD patients coexisting with diabetic nephropathy,^24^^,^^25^ which in line with our data in the population of T2DM coexisting with MAFLD. Collectively, our study added encouraging data to support the content that reduced omentin-1 levels were associated with unfavorable anthropometric and metabolic parameters in the population of T2DM and MAFLD alone or coexisted.

As a novel exercise-related hormone, irisin was found to decrease fat weight and serum TC and TG levels in diabetic mice while leading to greater phosphorylation of acetyl coenzyme A carboxylase-β in muscle tissue, elevated uncoupling protein 1 expression in fat tissue, and improved fatty acid oxidation in myocytes.^26^ In their study, the effects of irisin were achieved by activating the AMPK signaling pathway. In the context of MAFLD, high expression of irisin confers anti-inflammatory effects by disrupting the myeloid differentiation factor 2/toll-like receptor 4 pathway.^27^ Irisin was remarkably declined in MAFLD patients, and its supplement could affect the prooxidant-antioxidant balance in the liver.^28^ In our study, we also found levels of irisin decreased as anthropometric and metabolic parameters worsened in the study population of T2DM and MAFLD alone or coexisting. More similar to our study, Shanaki et al^29^ demonstrated lower levels of irisin in T2DM, MAFLD, and MAFLD+T2DM patients than the controls, while it was performed on Iranian people and the correlation analysis between irisin level and anthropometric and biochemical variables were limited due to several *r* values less than 0.4 and a small-scale sample size.

Several limitations should be pointed out. First, the case–control design may not sufficiently support the concept that the decreased levels of omentin-1 and irisin are probably causative factors related to MAFLD and diabetic parameters, which should be validated in further prospective and large-scale clinical investigations using longitudinal data. Second, the small sample size may bring caution to the results of correlation analysis between omentin-1, irisin, anthropometric, and biochemical parameters. Third, the degree of hepatosteatosis was not analyzed in this study; thus, the concept that omentin-1 and irisin play intrinsic roles in NAFLD is not established.

In conclusion, our findings demonstrate decreased levels of omentin-1 and irisin in T2DM coexisting with MAFLD compared to T2DM or MAFLD alone, suggesting the potential of omentin-1 and irisin in combination to assess the coexistence of T2DM and MAFLD. Considering omentin-1 and irisin as a combined detection for diagnosing T2DM coexisting with MAFLD showed greater performance than separate detection. These 2 of them can be integrated into a validated clinical risk prediction tool to enhance the assessment of coexisting patients. However, whether omentin-1 and irisin mediate the same pathway, thus potentiating their combined effects on the bidirectional association between T2DM and MAFLD, remains unknown. More large-scale longitudinal studies are needed to converge evidence linking omentin-1 and irisin to the pathogenesis of T2DM coexisting with MAFLD.

## Figures and Tables

**Table 1.** Anthropometric Characteristics and Clinical/Biochemical Profiles of Participants in T2DM, MAFLD, T2DM/MAFLD, and Control Groups

	T2DM (n = 80)	MAFLD (n = 62)	T2DM/MAFLD (n = 50)	Control (n = 80)
Age (year)	55.63 ± 8.24	53.58 ± 7.87	54.86 ± 6.75	52.80 ± 7.05
Sex (male)	43 (53.75%)	38 (61.29%)	28 (56.00%)	40 (50.00%)
BMI (kg/m^2^)	23.52 ± 2.76^ac^	26.63 ± 2.07	25.58 ± 2.58	22.67 ± 2.27^ac^
WHR	0.93 ± 0.03^ac^	0.96 ± 0.05^a^	0.99 ± 0.05	0.91 ± 0.04^ac^
SBP (mm Hg)	127.00 ± 8.02	125.50 ± 7.93^a^	130.10 ± 10.84	122.30 ± 7.61^ab^
DBP (mm Hg)	78.45 ± 8.26^a^	77.52 ± 8.10^a^	83.76 ± 6.38	75.83 ± 7.45^a^
FPG (mmol/L)	8.59 ± 0.92	5.55 ± 0.62^ab^	8.51 ± 1.03	5.13 ± 0.52^ab^
TC (mmol/L)	4.65 ± 0.86	4.73 ± 0.85	4.70 ± 0.85	4.49 ± 0.84
LDL-C (mmol/L)	2.70 ± 0.78	3.08 ± 0.84^b^	2.85 ± 0.89	2.61 ± 0.83^c^
HDL-C (mmol/L)	1.08 ± 0.22	1.14 ± 0.22	1.11 ± 0.23	1.34 ± 0.11^abc^
TG (mmol/L)	1.21 ± 0.33^ac^	1.90 ± 0.58	1.85 ± 0.40	1.04 ± 0.24^abc^
ALT (U/L)	18.00 (12.25, 26.75)^ac^	20.00 (14.75, 33.75)	22.00 (15.00, 32.5)	17.00 (10.00, 23.75)^ac^
AST (U/L)	16.00 (11.25, 21.00)^ac^	22.00 (14.00, 41.25)	21.00 (13.75, 37.75)	15.50 (9.00, 21.00)^ac^
GGT (U/L)	20.5 (13.25, 27.75)^ac^	28.5 (17.00, 40.50)	31.00 (15.75, 42.00)	19.00 (12.00, 24.75)^ac^
FINS (mLU/mL)	10.66 (8.07, 14.05)^a^	11.11 (8.66, 16.14)	15.33 (10.89, 18.00)	4.74 (4.14, 5.82)^abc^
HOMA-IR	3.89 (2.94, 5.58)	2.81 (2.07, 4.01)^ab^	5.43 (4.01, 7.06)	1.14 (0.94, 1.27)^abc^

When summarized as mean ± SD, quantitative variables were analyzed by one-way ANOVA with Tukey’s multiple comparisons test. When summarized as median with range (25% percentile, 75% percentile), quantitative variables were analyzed by Kruskal–Wallis test. ALP, alkaline phosphatase; ALT, alanine aminotransferase; AST aspartate aminotransferase; BMI, body mass index; DBP, diastolic blood pressure; FINS, fasting insulin; FPG, fasting plasma glucose; GGT, gamma glutamyl transferase; HDL-C high-density lipoprotein cholesterol; HOMA-IR, homeostasis model assessment of insulin resistance; LDL-C low-density lipoprotein cholesterol; MAFLD, metabolic-associated fatty liver disease; SBP, systolic blood pressure; T2DM, type 2 diabetes mellitus; TC, total cholesterol; TG triglyceride; WHR, waist circumference to hip circumference.

^a^*P* < .05 compared to T2DM/MAFLD group.

^b^*P* < .05 compared to T2DM group.

^c^*P* < .05 compared to MAFLD group.

**Table 2.** Pearson’s Correlations Between Circulating Omentin-1 and Irisin Levels, and Significant Anthropometric and Biochemical Variables Among T2DM, MAFLD, and T2DM/MAFLD Groups

Variable	T2DM (n = 80)		MAFLD (n = 62)		T2DM/MAFLD (n = 50)	
	Omentin-1	Irisin	Omentin-1	Irisin	Omentin-1	Irisin
BMI^#^	–	–	–	–	–	–
WHR^#^	–	–	−0.509	−0.427	−0.562	−0.482
SBP^#^	–	–	–	–	–	–
DBP^#^	–	–	–	–	–	–
FPG^#^	−0.408	−0.422				−0.542
TC^#^	–	–	–	–	–	–
LDL-C^#^	–	–	–	–	–	–
HDL-C^#^	0.434	–	0.534	–	0.644	–
TG^#^	–	–	–	–	−0.552	–
ALT	–	–	−0.451	–	–	–
AST^*^	–	–	−0.422	–	–	–
GGT^*^	–	–	–	–	–	–
FINS^*^	–	−0.437	–	−0.571	–	−0.410
HOMA-IR^*^	–	–	–	–	–	–

Only |*r* values| > 0.4 with *P* < .05 are presented.

ALP, alkaline phosphatase; ALT, alanine aminotransferase; AST aspartate aminotransferase; BMI, body mass index; DBP, diastolic blood pressure; FINS, fasting insulin; FPG, fasting plasma glucose; GGT, gamma glutamyl transferase; HDL-C high-density lipoprotein cholesterol; HOMA-IR, homeostasis model assessment of insulin resistance; LDL-C low-density lipoprotein cholesterol; MAFLD, metabolic-associated fatty liver disease; SBP, systolic blood pressure; T2DM, type 2 diabetes mellitus; TC, total cholesterol; TG triglyceride; WHR, waist circumference to hip circumference.

*Indicates *r* values yielded by Spearman’s correlation test.

^#^Indicates *r* values yielded by Pearson’s correlation test.

## Data Availability

Data sharing is not applicable to this article as no new data were created or analyzed in this study.
